# Possible recombination between two variants of concern in a COVID-19 patient

**DOI:** 10.1080/22221751.2022.2032375

**Published:** 2022-02-10

**Authors:** Yaqing He, Wentai Ma, Shengyuan Dang, Long Chen, Renli Zhang, Shujiang Mei, Xinyi Wei, Qiuying Lv, Bo Peng, Jiancheng Chen, Dongfeng Kong, Ying Sun, Xiujuan Tang, Weihua Wu, Zhigao Chen, Shimin Li, Jia Wan, Xuan Zou, Mingkun Li, Tiejian Feng, Lili Ren, Jianwei Wang

**Affiliations:** a Shenzhen Research Center for Communicable Disease Control and Prevention Chinese Academy of Medical Sciences, Shenzhen, People’s Republic of China; b Center for Disease Control and Prevention, Shenzhen, People’s Republic of China; c Key Laboratory of Genomic and Precision Medicine, Beijing Institute of Genomics, Chinese Academy of Sciences, and China National Center for Bioinformation, Beijing, People’s Republic of China; d University of Chinese Academy of Sciences, Beijing, People’s Republic of China; e National Health Commission of the People’s Republic of China Key Laboratory of Systems Biology of Pathogens and Christophe Mérieux Laboratory, Institute of Pathogen Biology, Chinese Academy of Medical Sciences & Peking Union Medical College, Beijing, People’s Republic of China; f Center for Excellence in Animal Evolution and Genetics, Chinese Academy of Sciences, Kunming, People’s Republic of China; g Key Laboratory of Respiratory Disease Pathogenomics, Chinese Academy of Medical Sciences and Peking Union Medical College, Beijing, People’s Republic of China

**Keywords:** SARS-CoV-2, intra-host single nucleotide variations, coinfection, recombination

## Abstract

We identified an individual who was coinfected with two SARS-CoV-2 variants of concern, the Beta and Delta variants. The ratio of the relative abundance between the two variants was maintained at 1:9 (Beta:Delta) in 14 days. Furthermore, possible evidence of recombinations in the *Orf1ab* and *Spike* genes was found.

## Introduction

Since the discovery of severe acute respiratory syndrome coronavirus 2 (SARS-CoV-2), there have been more than 1000 lineages identified during the global coronavirus disease 2019 (COVID-19) pandemic [1]. Some of the variants, which might have or have changed in infectivity, transmissibility, and antigenicity, were classified as variants of interest (VOI) and variants of concern (VOC). To date, a total of seven VOI and VOC variants have been identified [2].

With multiple variants circulating in the same place at the same time, coinfection with different SARS-CoV-2 variants becomes possible, which might give rise to new variants through viral homologous recombination [3–6]. Previous reports describing the genomic recombination of SARS-CoV-2 were based on the characterization of the mosaic structure in the population sequence data [3,6]. To date, no recombination event was observed at the individual level.

We have previously reported an outbreak of COVID-19 occurred on an international flight [7]. In this study, by analyzing the intra-host variation (iSNV) in the samples, we found that one patient who was previously thought to be infected with Delta variant was indeed coinfected by the Delta and Beta variants. Moreover, three genome regions showed unexpected frequency disruption, which might be caused by recombinations between two variants. The hypothesis was further supported by sequencing of tens of clones of the PCR product in the putative recombinant regions.

## Results

### Coinfection with two SARS-CoV-2 VOCs in one COVID-19 patient

An outbreak of COVID-19 occurred on a flight from Johannesburg, South Africa to Shenzhen, China (arrival date: 10 June 2021), 39 passengers on the plane got infected [7]. Four viral lineages, including two Delta strains (Delta-I and Delta-II), one Beta strain, and one C.1.2 strain, were identified (Supplementary Figure 1). Delta-I was the only strain observed in multiple cases in the outbreak, and showed low divergence among different cases (0-2 mutations), suggesting on-board transmission of this strain [7].

Case 49H had the highest number of iSNVs which was more than 30 in three consecutive samples (time-points: T1, June 22; T2, June 28; T3, July 3] (Supplementary Table 1). We noted that many of the iSNV positions were used to define the Beta variant and the Delta variant. Moreover, all 65 mutations possessed by the Delta-I strain and Beta strain can be detected in Case 49H, suggesting a coinfection in this case. Notably, Case 49H also had some low-level private mutations that were not observed in either the Delta-I or Beta strain (Supplementary Figure 2). Those mutations were not concurrently observed in the Delta-II, C.1.2 strains, or any viral genome deposited in public databases, hence were unlikely to represent the existence of a third strain, suggesting they may represent post-infection intra-host mutations.

Case 49E, who tested positive for SARS-CoV-2 on the arrival of the plane and sat next to Case 49H in the aircraft, was the only Beta strain-positive case identified in this outbreak. Meanwhile, all mutations possessed by Case 49E could be identified in Case 49H (Supplementary Figure 1). We also noted that Case 49E had close contact with Case 49H before boarding. Thus, the viruses in the two cases were likely to be derived from the same origin. As they had the same time of onset, it is unclear who was infected first. As for the origin of the Delta-I strain, Case 51C, who was suspected to be the index case of the outbreak and sat close to Case 49H, might transmit Delta-I strain to Case 49H whilst on the plane.

### Possible genomic recombination between the two variants

To further verify the coexistence of the two strains, seven genomic regions, which were shorter than 90 base pairs and included at least two mutation positions differing between the Beta and Delta-I strains, were selected for haplotype analysis. Of reads from the three time-point samples that spanned these seven regions, two major haplotypes were identified, corresponding to the Delta-I and Beta genome, respectively (Supplementary Table 2), thus supporting a scenario of coinfection.

**Figure 1.**
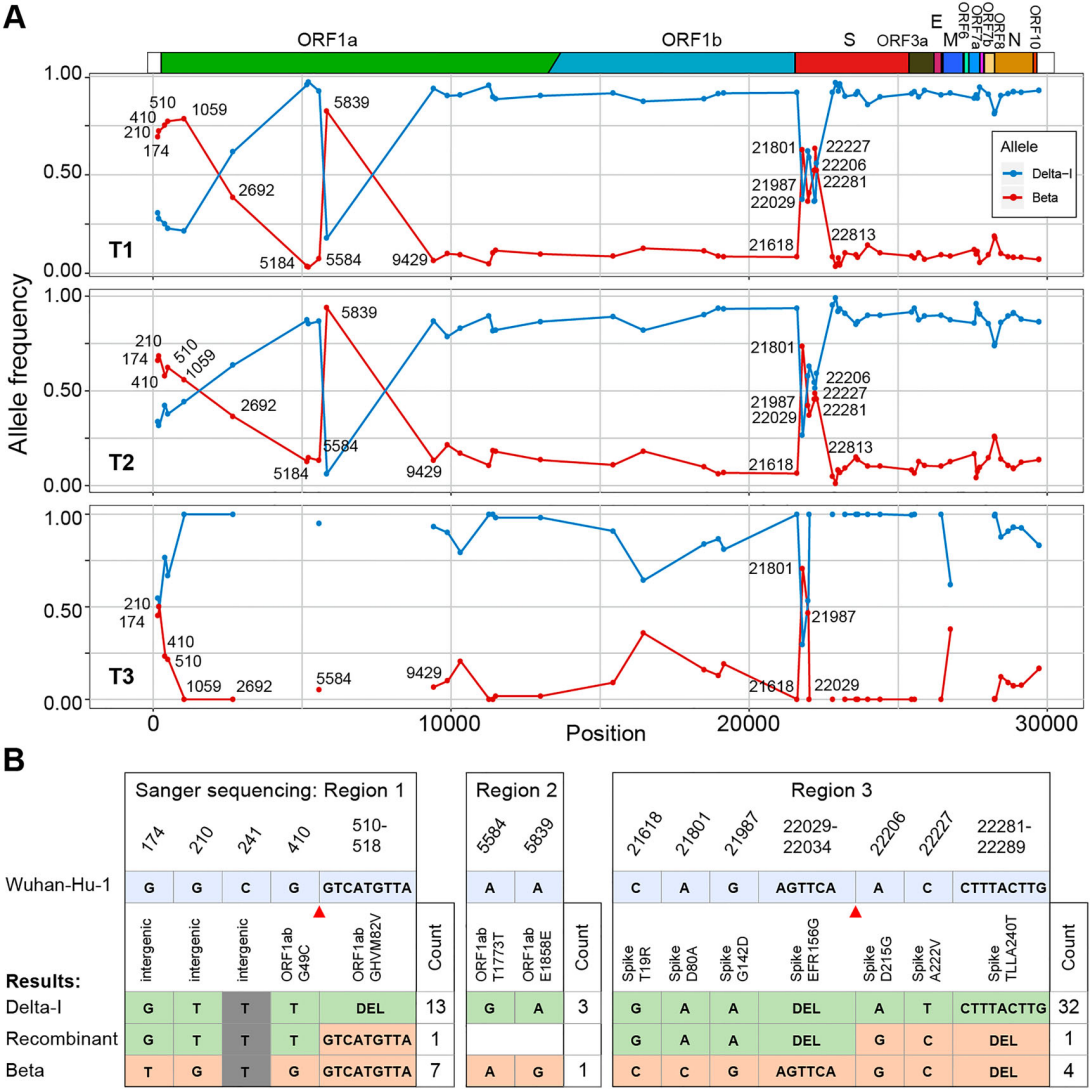
Signals of recombination in Case 49H. (A) The frequency of the Beta and Delta-I alleles in Case 49H. Only sites differing between the Delta-I genome and the Beta genome are shown. The frequency distribution are shown for different time-ponts (T1, T2 and T3). The missing sites on T3 were caused by limited sequencing depth in these regions. (B) The summary of haplotypes identified through TA clonal sequencing. Alleles that were identical to the Delta-I and Beta strain were labelled in different colours. Gray stands for non-informative sites (same allele possessed by two strains). The number of clones detected for each haplotype is labelled at the end of the sequences and the inferred recombination breakpoints are marked with red triangles.

We then calculated the ratio of these two variants in the sample by examining the mutant allele frequency at 60 positions that differed between the Delta-I strain and Beta strain. Notably, only the Beta and Delta-I alleles were detected at these positions, with the exception of two positions, where a third allele was detected with a frequency of 12% (Supplementary Table 3). At the T1 time-point, the ratio of Delta-I to Beta was 9 to 1 at most sites. Meanwhile, the ratio was approximately 1 to 1 or 1 to 3 at another 13 mutation sites, which were clustered into three regions of 174-2692, 5839, and 21801-22281, in the *Orf1ab* and *Spike* genes (Figure 1A). We found that the distribution of these discrepant positions was unlikely to be random (p-value = 2.9E-9, Wald–Wolfowitz runs test) [8]. Moreover, the result of 3SEQ and Bootscan recombination analysis also supported a mosaic genome structure at the consensus sequence level (p-value = 1.7E-3, Supplementary methods) [9,10]. Additionally, similar distributions were observed at the T2 and T3 time-points. The above results suggested the existence of additional haplotypes besides the Delta-I haplotype and the Beta haplotype in the body.

Sanger sequencing of the TA clones of the PCR products of the suspected regions was conducted using the leftover of T3 sample (the other two samples had no leftover, Supplementary Table 4). Sixty-two clones were successfully sequenced, two of them appeared to be chimeras of the two major haplotypes (Figure 1B). The one in region 1 was identical to the sequence of the Delta-I strain except that it lacks the deletion 510-518. Another chimeric read was found in region 3, whose first four alleles were identical to the Delta-I strain and the last three alleles were identical to the Beta strain at the variable sites. We speculated that the mosaic structure of these two sequences was likely to be derived through recombination between the Delta-I and Beta strains in vivo.

## Discussion

In this study, we observed the co-infection of the Delta and Beta variants in one case, and inter-lineage recombination events were suspected. The infected individuals who tested positive for SARS-CoV-2 on the arrival of the flight were quarantined separately. Therefore, the coinfection should occur prior to arrival in Shenzhen, either on the plane or before boarding.

Although the Delta variant was proved to be more transmissible and with higher replication efficiency compared to other variants [11], the proportion of the two variants was similar among samples collected in 14 days. Unfortunately, the viral load in the sample collected on the arrival of the plane was not enough for sequencing, making it unclear how the proportion was established and varied at the beginning of the coinfection. Nevertheless, the finding that different SARS-CoV-2 variants can remain coinfected in individuals for a period of time alerts us to the risk of recombination, which has not received widespread attention.

Recombination is common in coronaviruses. We identified three genomic regions where recombination may occur, 174-2692 and 5839 in the *Orf1ab* gene and 21801-22281 in the *Spike* gene. The region of 21801-22281 had been proposed as a putative recombination region between the progenitor of SARS-CoV-2 and Bat-SL-CoV, and the other two regions also exhibited evidence of recombination [12,13]. Possible recombinant sequences of two parental strains were identified in Sanger sequencing.

Although PCR amplification was performed before the sequencing, which may result in random recombinant reads [14], it cannot explain the iSNV pattern we observed in the study, as the frequency disrupted positions were distributed non-randomly across the genome. Moreover, PCR amplification was routinely conducted in SARS-CoV-2 genome sequencing but the same pattern was not reported in previous studies, including those investigated coinfections [4].

It has been reported that mutations may cause enrichment bias for different variants[15]. However, the recombination signal in our study is unlikely to be explained by the enrichment bias because 1) the sequencing depth was similar among different samples, including those belonging to different lineages (Supplementary Figure 3); 2) based on the enrichment bias we estimated, two of three hypothetical recombination regions were Beta-enriched (5839, 21801-22281), whereas no enrichment bias was observed in the 174-2692 region (Supplementary Figure 4); 3) the ratio of frequency between the two variants in the three hypothetical recombination regions showed the largest deviation from the expected value by considering the enrichment bias (Supplementary Figure 5).

We noted that other studies have also identified coinfections and recombination between different SARS-CoV-2 variants [3]. Thus, the events observed in our study may not be rare, especially considering that there are hundreds of variants circulating in the population and over 30 million new infections in the past 28 days (as of Jan 11, 2022). Non-medical interventions, such as the use of masks and social distancing, should be continuously implemented, and the patients infected with different virus variants should be isolated separately to prevent the emerging of new recombinant viruses.

## Supplementary Material

Supplemental Material

Supplemental Material

## References

[1] Rambaut A, Holmes EC, O'Toole Á, et al. A dynamic nomenclature proposal for SARS-CoV-2 lineages to assist genomic epidemiology. Nat Microbiol. 2020 Nov;5(11):1403–1407. PubMed PMID: 32669681. 32669681 10.1038/s41564-020-0770-5 PMC7610519

[2] WHO|World Health Organization. Available from: www.who.int.

[3] Jackson B, Boni MF, Bull MJ, et al. Generation and transmission of interlineage recombinants in the SARS-CoV-2 pandemic. Cell. 2021 Sep 30;184(20):5179–5188.e8. doi:10.1016/j.cell.2021.08.014. PubMed PMID: 34499854. 34499854 PMC8367733

[4] Tonkin-Hill G, Martincorena I, Amato R, et al. Patterns of within-host genetic diversity in SARS-CoV-2. eLife. 2021 Aug 13;10; doi:10.7554/eLife.66857. PubMed PMID: 34387545. PMC8363274 34387545

[5] Varabyou A, Pockrandt C, Salzberg SL, et al. Rapid detection of inter-clade recombination in SARS-CoV-2 with Bolotie. Genetics. 2021 May 13;218(3), doi:10.1093/genetics/iyab074. PubMed PMID: 33983397. PMC8194586 33983397

[6] Fischer W, Giorgi EE, Chakraborty S, et al. HIV-1 and SARS-CoV-2: Patterns in the evolution of two pandemic pathogens. Cell Host Microbe. 2021 Jul 14;29(7):1093–1110. doi:10.1016/j.chom.2021.05.012. PubMed PMID: 34242582. 34242582 PMC8173590

[7] Lv Q, Kong D, He Y, et al. A SARS-CoV-2 Delta variant outbreak on airplane: Vaccinated air passengers are more protected than unvaccinated. J Travel Med. 2021 Oct 5;28(8), doi:10.1093/jtm/taab161. PubMed PMID: 34609488. PMC8522384 34609488

[8] Song H, Giorgi EE, Ganusov VV, et al. Tracking HIV-1 recombination to resolve its contribution to HIV-1 evolution in natural infection. Nat Commun. 2018 May 15;9(1):1928, doi:10.1038/s41467-018-04217-5. PubMed PMID: 29765018. 29765018 PMC5954121

[9] Lam HM, Ratmann O, Boni MF. Improved Algorithmic Complexity for the 3SEQ Recombination Detection Algorithm. Mol Biol Evol. 2018 Jan 1;35(1):247–251. doi:10.1093/molbev/msx263. PubMed PMID: 29029186. 29029186 PMC5850291

[10] Lole KS, Bollinger RC, Paranjape RS, et al. Full-length human immunodeficiency virus type 1 genomes from subtype C-infected seroconverters in India, with evidence of intersubtype recombination. J Virol. 1999 Jan;73(1):152–160. doi:10.1128/jvi.73.1.152-160.1999. PubMed PMID: 9847317. 9847317 PMC103818

[11] Wang Y, Chen R, Hu F, et al. Transmission, viral kinetics and clinical characteristics of the emergent SARS-CoV-2 Delta VOC in Guangzhou. China. EClinMed. 2021 Oct;40:101129), doi:10.1016/j.eclinm.2021.101129. PubMed PMID: 34541481. PMC8435265 34541481

[12] Zhu Z, Meng K, Meng G. Genomic recombination events may reveal the evolution of coronavirus and the origin of SARS-CoV-2. Sci Rep. 2020 Dec 10;10(1):21617), doi:10.1038/s41598-020-78703-6. PubMed PMID: 33303849. 33303849 PMC7728743

[13] Haddad D, John SE, Mohammad A, et al. SARS-CoV-2: Possible recombination and emergence of potentially more virulent strains. PloS one. 2021;16(5):e0251368), doi:10.1371/journal.pone.0251368. PubMed PMID: 34033650. 34033650 PMC8148317

[14] Meyerhans A, Vartanian JP, Wain-Hobson S. DNA recombination during PCR. Nucleic Acids Res. 1990 Apr 11;18(7):1687–1691. doi:10.1093/nar/18.7.1687. PubMed PMID: 2186361. 2186361 PMC330584

[15] Ma W, Yang J, Fu H, et al. Genomic perspectives on the emerging SARS-CoV-2 omicron variant. Genomics Proteomics Bioinf. 2022 Jan 13; doi:10.1016/j.gpb.2022.01.001. PubMed PMID: 35033679. PMC8791331 35033679

