# Supplementary material for: Possible recombination between two variants of concern in a COVID-19 patient
Source: Emerg Microbes Infect. 2022 Feb 10;11(1):552–5. doi: 10.1080/22221751.2022.2032375 (PMC8843165; doi:10.1080/22221751.2022.2032375)
Supplement: Supplemental Material [file TEMI_A_2032375_SM2775.docx]

**Possible recombination between two variants of concern in a COVID-19 patient**

**Methods**

**Subjects and samples**

The nasopharyngeal swabs were collected from passengers on flight CA868 on arrival and during the quarantine. All passengers gave their oral and written informed consent. The study was approved by the Ethics Committee of Shenzhen Center for Disease Control and Prevention. All samples were screened by SARS-CoV-2 rapid detection kits (DAAN gene and BioGerm, China).

**Nucleic acid extraction and sequencing**

A total of 200μl samples were used for nucleic acids extraction by High Pure Viral RNA Kit (Roche, Germany). SARS-CoV-2 nucleic acids were enriched by ULSEN® 2019-nCoV Whole Genome Kit (Beijing MicroFuture, China), and the sequencing libraries were prepared by the Illumina Nextera® XT Library Prep Kit (Illumina, USA), which was sequenced on the MiSeq platform (Illumina, USA) with a read length of 150 bp (paired-end mode).

**Sanger sequencing**

Three putative recombinant regions were amplified using the primers described in Supplementary Table 2. Hieff Clone® Zero TOPO-TA Cloning Kit (HB180280, Yeasen, China) was used to clone the target fragments. UNlQ-10 Column Yeast Plasmid Preps Kit (SK8191, Yeasen, China) was used to extract plasmid DNA. Plasmids were cut by PstI single enzyme. The size of the fragments was determined by agarose gel electrophoresis. Finally, ABI 3730 DNA analyzer was used to do the DNA sequencing. The sequencing results were visualized by Chromas (V 2.6.5).

**SARS-CoV-2 genome sequence analysis**

The quality control and adaptor trimming were performed by FASTP (-l 50 --cut_tail –cut_tail_mean_quality 20) [1]. Taxonomic classification was done by Kraken v2.0.8 [2]. Reads classified as *Cornidovirineae* were mapped to the SARS-CoV-2 reference genome (Wuhan-Hu-1, GenBank: MN908947.3) using BWA-MEM software [3]. Minimap2 was used to search for any potentially existing long deletions [4]. Deduplication was performed with Picard toolkit (1.119) [5]. BamUtil trimBam was used to remove 10 bp on both ends of mapped read to avoid false-positive iSNVs associated with misalignment. The nucleotide mpileup file and the read count file were generated by SAMtools and Varscan2 [6, 7]. The consensus sequence was identified using the following criteria, 1) Depth ≥ 5 folds; 2) Frequency of the major allele ≥ 70%; 3) Major allele was supported by reads mapped to both strands. The virus lineage was assigned by the Pango web server (3.1.11) [8].

**Intra-host nucleotide variation analysis**

Intra-host nucleotide variation (iSNVs) was called at positions with a sequencing depth no less than ten-fold. Only mutation with allele frequency ≥ 10% and supported by at least three reads on each strand were considered as iSNVs. The mutant allele frequency was calculated based on the readcounts file generated by Varscan2 [6].

**Haplotype analysis**

Reads mapped to the questioned regions were extracted by Samtools. An in-house script was used to retrieve the allele at the mutant positions to construct the haplotype. Only reads spanning over the region and an addition 5 bp in both directions were used to estimate the fraction of haplotype in the region.

**Wald-Wolfowitz runs test**

The Wald-Wolfowitz runs test checks a randomness hypothesis for a two-valued data sequence, and can also be used for recombination detection [9]. To check whether the distribution of the discrepant positions from Case 49H were caused by randomness e.g. sequencing error, the 60 mutation sites that differ between the Delta-I genome and the Beta genome at T1 time-point were separated into two types: the Delta-I similar (Delta-I allele frequency >= 70%) and the Delta-I dissimilar (Delta-I allele frequency < 70%). The two types of sites formed six runs, three runs of the Delta-I similar (5184~5584, 9429~21618, 22813~29743), and three runs of the Delta-I dissimilar (174~2692, 5839, 21801~22281). Then, the mean and variance were calculated based on the formulae of Wald-Wolfowitz runs test (N = 60, N_+_ = 13, N_-_ = 47, r = 6). Finally, the p-value was converted from the z-score.

Mean: $\mu=\frac{N_{+}N_{-}}{N}+1$

Variance: $\sigma=\sqrt{\frac{2N_{+}N_{-}(2N_{+}N_{-}-N)}{N^{2}(N-1)}}$

1. score: $Z =\frac{r - \mu}{\sigma}$

**Test for mosaic structure at the consensus sequence level**

Recombination analysis was performed by 3SEQ v1.7 and Bootscan using the consensus sequences of Cases 51C, 49E, and 49H (at T1 time point) [10, 11].

**Supplementary Figures**


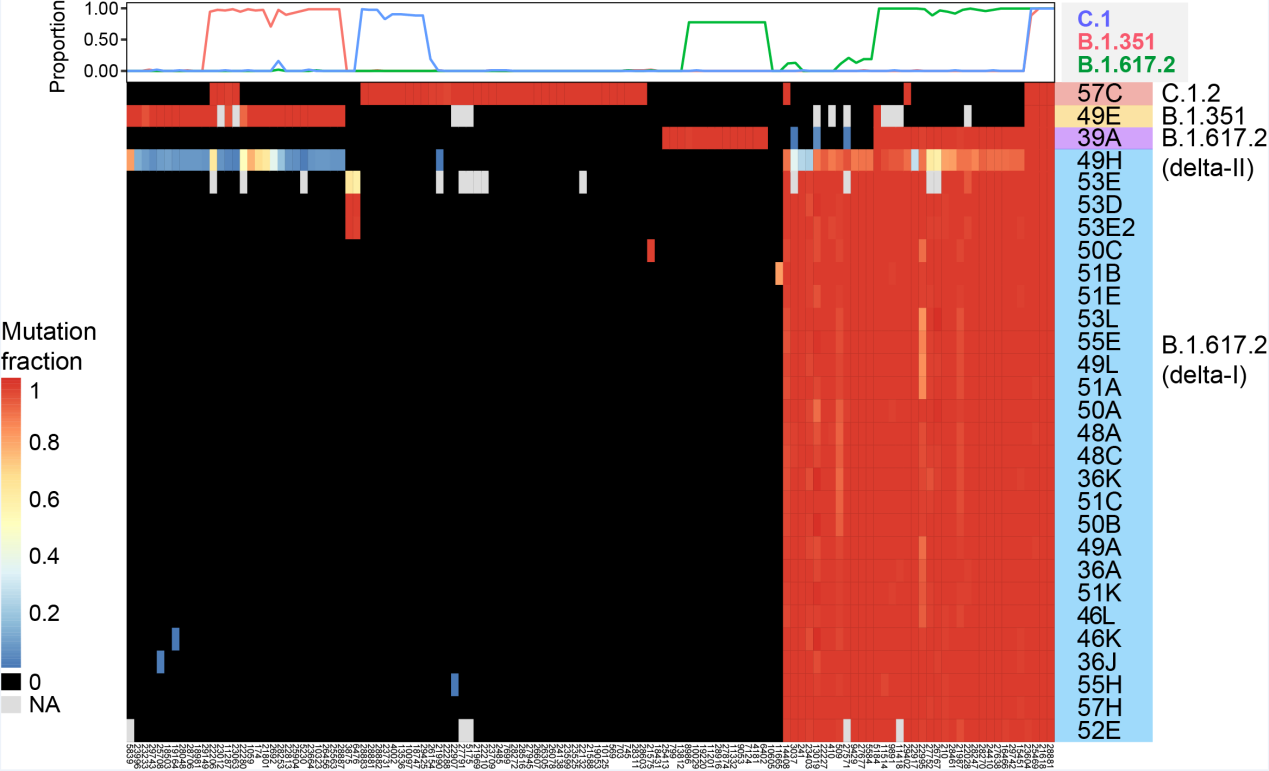


**Supplementary Figure 1.** **The mutation fraction heatmap of all genomic mutations identified from 29 patients.** The 49H in the figure represents its T1 time-point sample. The line chart on the top shows the frequency of the mutations in samples belonging to different lineages in the public database on 8 Aug 2021.


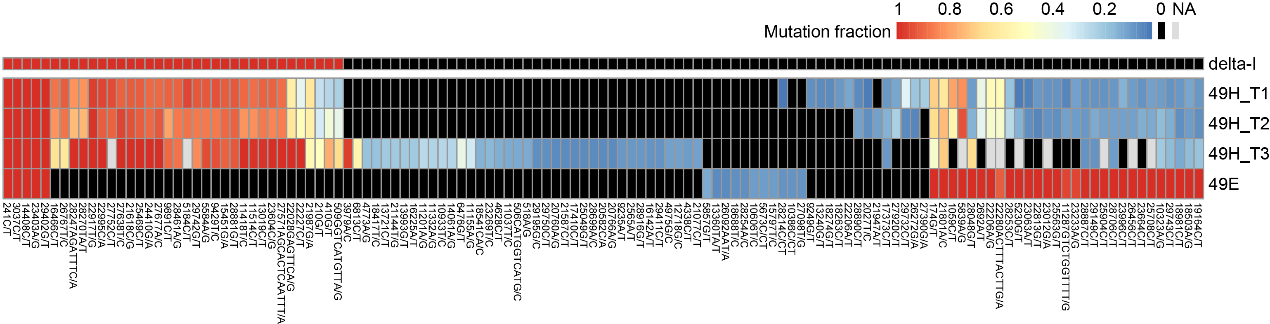


**Supplementary Figure 2. The frequency of mutations (including intra-host variations) that were identified in Case 49H and 49E.** The schematic bar showed the mutation in the Delta-I strain.


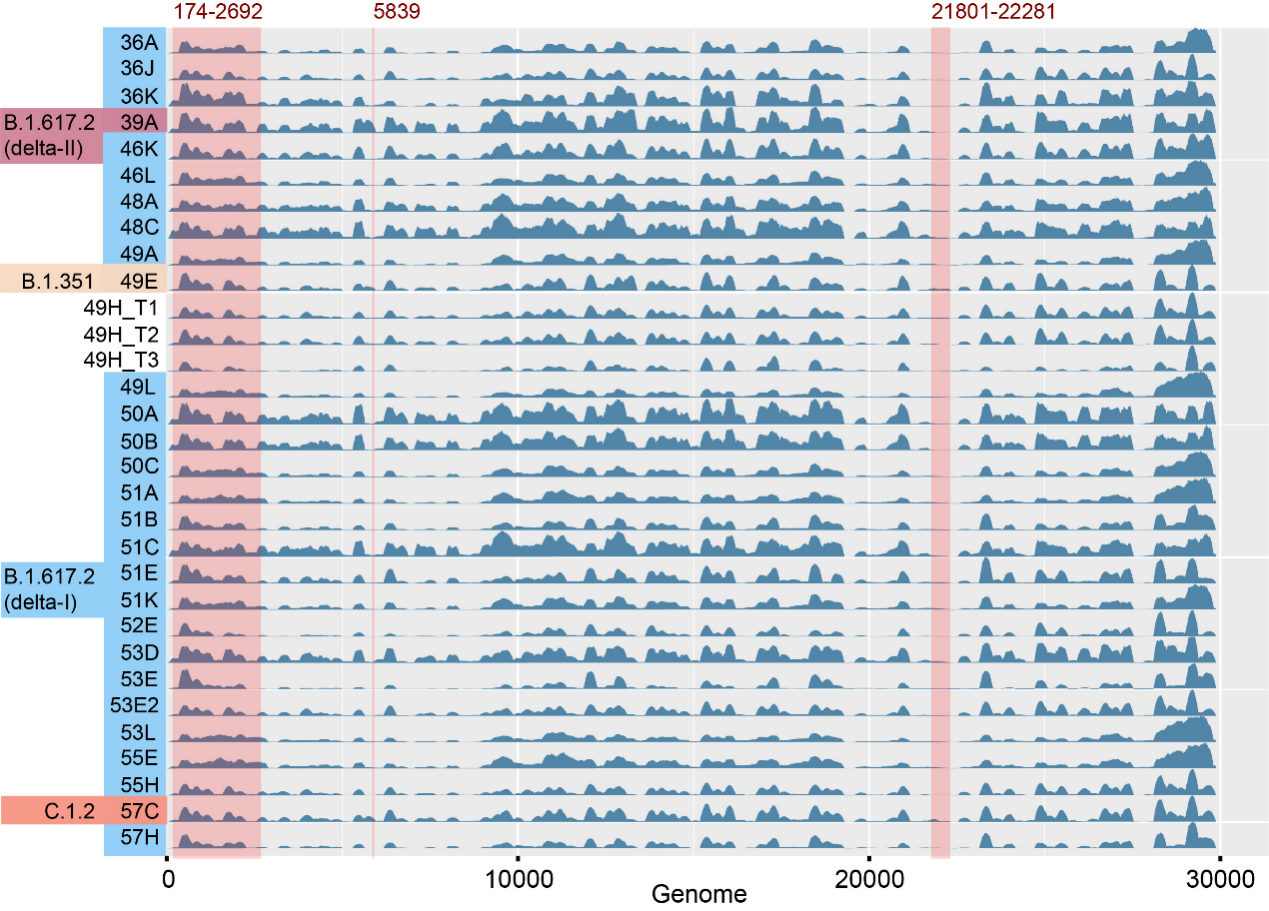


**Supplementary Figure 3. The normalized sequencing depth distribution of 31 sequenced samples.** Sequencing depths were normalized by the maximum depth in the sample. The three hypothetical recombination regions are highlighted in red. The ID of the samples belonging to different lineages were highlighted in different colors.


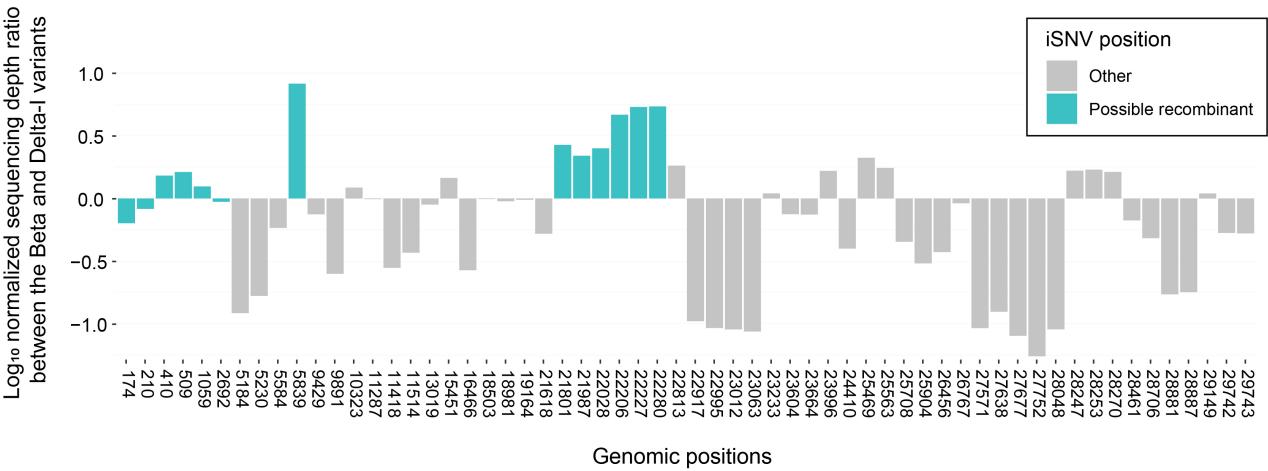


**Supplementary Figure 4. The enrichment bias of the Beta and Delta-I strains.** The 60 sites differing between the Delta-I genome and the Beta genome are shown. Each bar represents the normalized sequencing depth ratio (Beta/Delta) for that position. Positions located in the hypothetical recombination regions are filled in cyan.


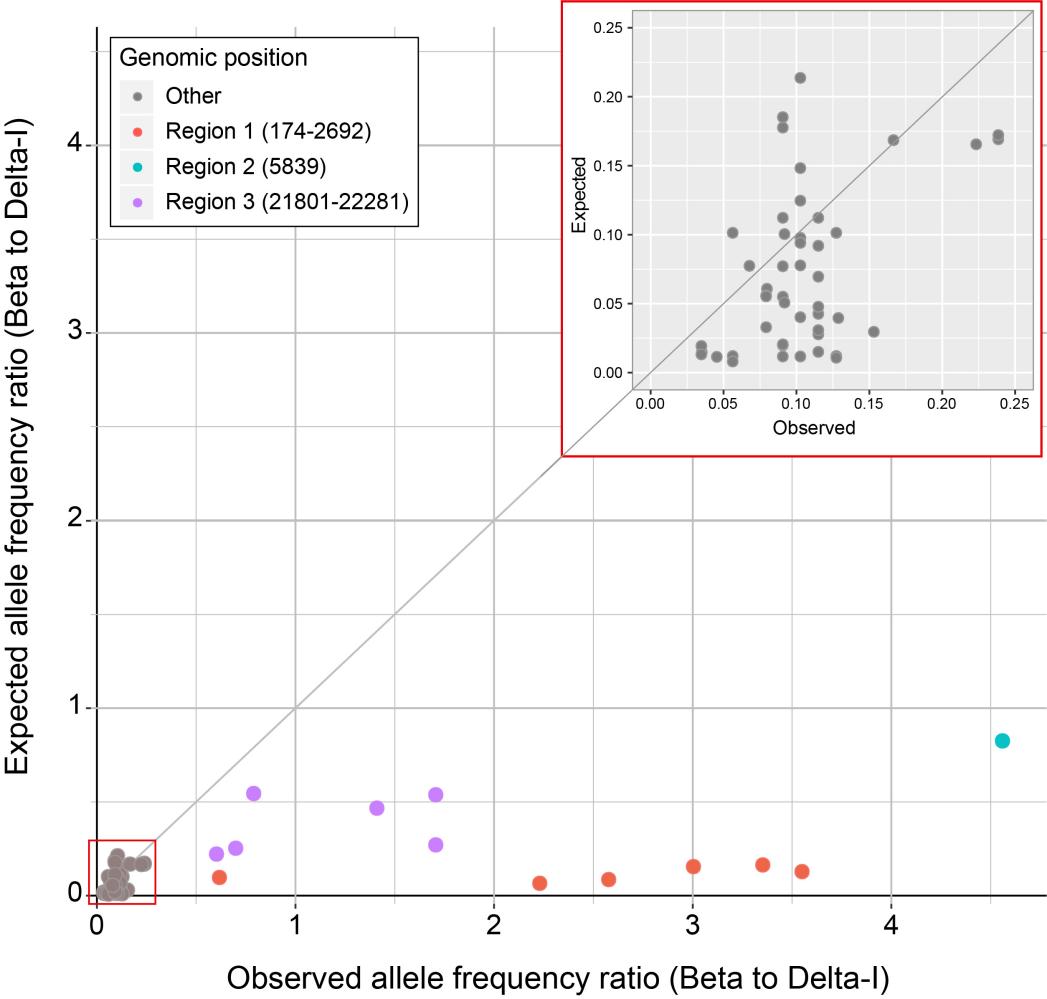


**Supplementary Figure 5. The correlation between the expected allele frequency ratio and the observed allele frequency ratio in Case 49H at the T1 time-point.** Each dot represents a genomic position differing between the Delta-I genome and the Beta genome. The expected allele frequency was calculated by multiplying the median allele frequency ratio of 47 variant sites that located outside the hypothetical recombination regions by the enrichment bias (normalized sequencing depth ratio between Beta and Delta). The upper right inset shows an enlarged version of the area at the lower left.

**Supplementary Tables**

**Supplementary Table 1.** The sampling timepoint and Ct value of all samples collected from Case 49H

| Sampling Time | Ct value | | Sample type | Sample name |
| --- | --- | --- | --- | --- |
|  | ORF1ab | N |  |  |
| 10-Jun | 35 | 35 | nasopharyngeal swab | Unsequenced |
| 10-Jun | 38.5 | Negative | nasopharyngeal swab | Unsequenced |
| 11-Jun | 33 | 35 | nasopharyngeal swab | Unsequenced |
| 11-Jun | Negative | Negative | nasopharyngeal swab | Unsequenced |
| 11-Jun | Negative | Negative | blood | Unsequenced |
| 22-Jun | 35 | 35 | nasopharyngeal swab | T1 time-point |
| 28-Jun | 35 | 35 | nasopharyngeal swab | T2 time-point |
| 30-Jun | Negative | 37 | nasopharyngeal swab | Unsequenced |
| 2-Jul | Negative | Negative | nasopharyngeal swab | Unsequenced |
| 3-Jul | 31 | 32 | nasopharyngeal swab | T3 time-point |
| 4-Jul | Negative | 38 | nasopharyngeal swab | Unsequenced |
| 5-Jul | Negative | Negative | nasopharyngeal swab | Unsequenced |
| 6-Jul | Negative | Negative | nasopharyngeal swab | Unsequenced |
| 9-Jul | Negative | 38 | nasopharyngeal swab | Unsequenced |
| 12-Jul | Negative | Negative | nasopharyngeal swab | Unsequenced |
| 15-Jul | Negative | Negative | nasopharyngeal swab | Unsequenced |
| 19-Jul | Negative | Negative | nasopharyngeal swab | Unsequenced |
| 24-Jul | Negative | Negative | nasopharyngeal swab | Unsequenced |
| 30-Jul | Negative | Negative | nasopharyngeal swab | Unsequenced |
| 1-Aug | Negative | Negative | nasopharyngeal swab | Unsequenced |
| 2-Aug | Negative | Negative | nasopharyngeal swab | Unsequenced |

**Supplementary Table 2.** The haplotype distribution in seven genomic regions in Case 49H

| **Genome position** | **49E (Beta)** | **51C(Delta-I)** | **Haplotype** | **Reads number (proportion)^a^** | | |
| --- | --- | --- | --- | --- | --- | --- |
|  |  |  |  | **T1** | **T2** | **T3** |
| 174, 210, 241 | T, G, T | G, T, T | T, G, T | 369(0.69) | 403(0.66) | 14(0.5) |
|  |  |  | G, T, T | 160(0.3) | 208(0.34) | 13(0.46) |
| 5184, 5230 | C, T | T, G | T, G | 165(0.98) | 154(0.88) | No Coverage |
|  |  |  | C, T | 4(0.02) | 21(0.12) |  |
| 22206, 22227, 22281 | G, C, DEL | A, T, CTTTACTTG | G, C, DEL | 45(0.51) | 26(0.39) | NA |
|  |  |  | A, T, CTTTACTTG | 32(0.36) | 38(0.57) | 2(1) |
|  |  |  | T, C, DEL | 12(0.13) | 0 | 0 |
| 23604, 23664 | C,T | G,C | G,C | 738(0.91) | 839(0.86) | 14(1) |
|  |  |  | C,T | 69(0.08) | 133(0.13) | 0 |
| 28248, 28253, 28271 | GATTTC, T, A | DEL, C, DEL | DEL, C, DEL | 9297(0.8) | 7444(0.73) | 1065(0.99) |
|  |  |  | GATTTC, T, A | 2246(0.19) | 2748(0.27) | 0 |
| 28881, 28887 | G, T | T, C | T, C | 3605(0.92) | 2585(0.91) | 67(0.91) |
|  |  |  | G, T | 313(0.08) | 259(0.09) | 6(0.08) |
| 29742, 29743 | G,T | T,C | T,C | 4879(0.92) | 4302(0.81) | 1218(0.83) |
|  |  |  | G,T | 365(0.07) | 711(0.13) | 246(0.17) |

^a^ Only haplotypes with frequency >=1% and supported by at least two reads are shown.

**Supplementary Table 4. Primers used to amplify the putative recombinant genomic regions**

| Primer | Sequences | Covered regions |
| --- | --- | --- |
| Primer1- Forward | CCTTCCCAGGTAACAAACC | 15-664 |
| Primer1- Reverse | GCCACCAGCTCCTTTATTAC |  |
| Primer2- Forward | GCACTTATCTTAGCCTACTGT | 5399-6173 |
| Primer2- Reverse | TAGCCACCACATCACCAT |  |
| Primer3- Forward | GCCACTAGTCTCTAGTCAGT | 21586-22437 |
| Primer3- Reverse | GCACAGTCTACAGCATCT |  |

**Reference**

1. Chen S, Zhou Y, Chen Y, et al. fastp: an ultra-fast all-in-one FASTQ preprocessor. Bioinformatics (Oxford, England). 2018 Sep 1;34(17):i884-i890. doi: 10.1093/bioinformatics/bty560. PubMed PMID: 30423086.

2. Wood DE, Lu J, Langmead B. Improved metagenomic analysis with Kraken 2. Genome biology. 2019 Nov 28;20(1):257. doi: 10.1186/s13059-019-1891-0. PubMed PMID: 31779668.

3. Li H, Durbin R. Fast and accurate short read alignment with Burrows-Wheeler transform. Bioinformatics (Oxford, England). 2009 Jul 15;25(14):1754-60. doi: 10.1093/bioinformatics/btp324. PubMed PMID: 19451168.

4. Li H. Minimap2: pairwise alignment for nucleotide sequences. Bioinformatics (Oxford, England). 2018 Sep 15;34(18):3094-3100. doi: 10.1093/bioinformatics/bty191. PubMed PMID: 29750242.

5. McKenna A, Hanna M, Banks E, et al. The Genome Analysis Toolkit: a MapReduce framework for analyzing next-generation DNA sequencing data. Genome research. 2010 Sep;20(9):1297-303. doi: 10.1101/gr.107524.110. PubMed PMID: 20644199.

6. Koboldt DC, Zhang Q, Larson DE, et al. VarScan 2: somatic mutation and copy number alteration discovery in cancer by exome sequencing. Genome research. 2012 Mar;22(3):568-76. doi: 10.1101/gr.129684.111. PubMed PMID: 22300766.

7. Li H, Handsaker B, Wysoker A, et al. The Sequence Alignment/Map format and SAMtools. Bioinformatics (Oxford, England). 2009 Aug 15;25(16):2078-9. doi: 10.1093/bioinformatics/btp352. PubMed PMID: 19505943.

8. Rambaut A, Holmes EC, O'Toole Á, et al. A dynamic nomenclature proposal for SARS-CoV-2 lineages to assist genomic epidemiology. Nature microbiology. 2020 Nov;5(11):1403-1407. doi: 10.1038/s41564-020-0770-5. PubMed PMID: 32669681.

9. Song H, Giorgi EE, Ganusov VV, et al. Tracking HIV-1 recombination to resolve its contribution to HIV-1 evolution in natural infection. Nature communications. 2018 May 15;9(1):1928. doi: 10.1038/s41467-018-04217-5. PubMed PMID: 29765018.

10. Lam HM, Ratmann O, Boni MF. Improved Algorithmic Complexity for the 3SEQ Recombination Detection Algorithm. Molecular biology and evolution. 2018 Jan 1;35(1):247-251. doi: 10.1093/molbev/msx263. PubMed PMID: 29029186.

11. Lole KS, Bollinger RC, Paranjape RS, et al. Full-length human immunodeficiency virus type 1 genomes from subtype C-infected seroconverters in India, with evidence of intersubtype recombination. Journal of virology. 1999 Jan;73(1):152-60. doi: 10.1128/jvi.73.1.152-160.1999. PubMed PMID: 9847317.
